# Detection of secreted antimicrobial peptides isolated from cell-free culture supernatant of *Paenibacillus alvei* AN5

**DOI:** 10.1007/s10295-013-1259-5

**Published:** 2013-03-19

**Authors:** Bassam Alkotaini, Nurina Anuar, Abdul Amir Hassan Kadhum, Asmahani Azira Abdu Sani

**Affiliations:** 1 Department of Chemical and Process Engineering, Faculty of Engineering and Built Environment, Universiti Kebangsaan Malaysia, 43600 Bangi, Selangor Malaysia; 2 Proteomic Laboratory, Malaysia Genome Institute, Ministry of Science, Technology and Innovation, 43000 Jalan Bangi, Selangor Malaysia

**Keywords:** *Paenibacillus alvei* AN5, Antimicrobial peptide AN5-1, LC/ESI–MS, Action mode, Stability

## Abstract

An antimicrobial substance produced by the *Paenibacillus alvei* strain AN5 was detected in fermentation broth. Subsequently, cell-free culture supernatant (CFCS) was obtained by medium centrifugation and filtration, and its antimicrobial activity was tested. This showed a broad inhibitory spectrum against both Gram-positive and -negative bacterial strains. The CFCS was then purified and subjected to SDS-PAGE and infrared spectroscopy, which indicated the proteinaceous nature of the antimicrobial compound. Some de novo sequencing using an automatic Q-TOF premier system determined the amino acid sequence of the purified antimicrobial peptide as Y-S-K-S-L-P-L-S-V-L-N-P (1,316 Da). The novel peptide was designated as peptide AN5-1. Its mode of action was bactericidal, inducing cell lysis in *E. coli* ATCC 29522 and *S. aureus*, and non-cell lysis in both *S. marcescens* and *B. cereus* ATCC 14579. Peptide AN5-1 displayed stability at a wide range of pH values (2–12) and remained active after exposure to high temperatures (100 °C). It also maintained its antimicrobial activity after incubation with chemicals such as SDS, urea and EDTA.

## Introduction

Due to the increasing numbers of resistant pathogenic bacteria and side effects caused by existing antibiotics, new antimicrobial compounds with effective properties are needed [9]. Antimicrobial peptides are attracting increasing interest as a potential substitute. The importance of antimicrobial peptides has been appreciated in the last 20 years due to pathogenic bacterial resistance against existing antibiotics [24].

Antimicrobial peptides are small proteins occurring in living organisms and are produced as defense molecules against pathogens such as bacteria. Therefore, it is considered the first line of defense in invaded eukaryotic and prokaryotic cells [24]. Their mode of action varies between peptides. Three factors play a major role in determining the mode of action: the net positive charge on the surface, its 3-D amphipathic structure and the selective disruption points on the target cell membrane [17]. These actions were studied by Hallock et al. [10] based on solid-state NMR, which showed an interaction between antimicrobial peptides and membranes, leading to membrane disruption. However, in terms of medical and food applications, antimicrobial peptides have no toxic effects on mammalian cells [20], making them a promising candidate for further studies.

In 1925, the first antimicrobial peptide was discovered from Gram-negative bacteria, *Escherichia coli*, known ascolicins. This was followed by the discovery of nisin in 1928, which was an antimicrobial peptide produced by the Gram-positive *Streptococcus lactis* (formerly known as *Lactococcus lactis*) [12]. Since then, several Gram-positive bacteria, in particular soil bacteria bacilli, have been reported to produce antimicrobial agents such as bacitracin, lichenin and cerein, which are produced by *Bacillus subtilis*, *Bacillus licheniformis* and *Bacillus cereus*, respectively [7, 19, 23]. Production of antimicrobial peptides is considered an unstable bacterial characteristic because it changes according to growth conditions. A tradeoff between metabolism and gene expression affects antimicrobial peptide biosynthesis during bacterial growth [26].

*Paenibacillus alvei* are Gram-positive, rod-shaped, motile, spore-forming and catalase-positive bacteria [22]. The first report of antimicrobial peptide production from this bacteria was by Anandaraj et al. [1], who isolated a strain from fermented tomato fruit and detected two antimicrobial peptides, Paenibacillin P and Paenibacillin N. Other species of *Paenibacillus* have been reported as antimicrobial peptide producers too. For example, polymyxins, LI-F complex, saltavalin and gatavalin are produced by different strains of *Paenibacillus polymyxa* [25]. This study aimed to isolate an antimicrobial peptide from the cell-free culture supernatant (CFCS) of *P. alvei* AN5, screen its antimicrobial activity and identify the amino acid sequence of the antimicrobial peptide.

## Materials and methods

### Microorganisms

*Paenibacillus alvei* AN5 with a 16S rRNA gene accession number of JQ868768 at GenBank was obtained from the Biochemical Engineering Laboratory, Faculty of Engineering and Built Environment, Universiti Kebangsaan Malaysia. It was grown in nutrient broth (NB) (Oxoid limited, UK) at 30 °C, under agitation at 150 rpm for 24 h. The test strains used in this study (Table 1) were obtained from the American Type Culture Collection (ATCC) and Biochemical Engineering Laboratory, Faculty of Engineering and Built Environment, UKM. All bacterial strains were grown in tryptic soy broth (TSB) (Difco, France) and NB at 37 °C, under agitation at 150 rpm for 24 h.

**Table 1:** Antimicrobial activity of cell free culture supernatant (CFCS) from *P. alvei* AN5 and the MICs of the synthesized antimicrobial peptide AN5-1 against test strains

Bacterial test strains	Inhibition zones of CFCS	MICs (μg ml^−1^) of AN5-1
*Staphylococcus aureus* ^a^	+++	64
*Streptococcus agalactiae* ATCC 12386	–	–
*Staphylococcus epidermidis*	–	–
*Lactobacillus plantarum* ATCC 8014	+++	32
*Lactococcus lactis* ATCC 11454	+	>128
*Bacillus subtilis*	+++	32
*Bacillus cereus* ATCC 14579	++	64
*Bacillus anthracis*	+	128
*Bacillus licheniformis*	+	>128
*Bacillus flexus* ^a^	++++	32
MRSA	–	–
*Escherichia coli* ATCC 25922	+++	8
*Escherichia coli*	+++	8
*Salmonella enteritidis* ATCC 13076	++++	4
*Pseudomonas aeruginosa*	–	–
*Enterobacter spp.*	+++	16
*Serratia marcescens* ^a^	++++	32

–, no inhibition zones; +, zone diameter 1–11 mm; ++, zone diameter 12–16 mm; +++, zone diameter 16–21 mm. ++++ zone diameter ≥22 mm

^a^Test strains are incubated in NA

### Production of the antimicrobial compound

*Paenibacillus alvei* AN5 was grown in NB at 30 °C for 24 h. The resulting culture was used to inoculate a 2 l flask containing 500 ml of modified Landy medium consisting of 20 g l^−1^ glucose, 2 g l^−1^ yeast extract, 5.0 g l^−1^ l-glutamic acid, 2 g l^−1^ KH_2_PO_4_, 0.16 mg l^−1^ CuSO_4_, 0.5 g l^−1^ MgSO_4_·7H_2_O, 0.15 mg l^−1^ FeSO_4_, 0.5 g l^−1^ KCl, 4 g l^−1^ NaCl, 1 g l^−1^ (NH_4_)NO_2_, 1 g l^−1^ NH_4_SO_4_ and 45.0 mg l^−1^ MnSO_4_·H_2_O, pH 7. The sample was incubated at 30 °C, under agitation at 150 rpm for 24 h. The CFCS was obtained at 2-h intervals by culture centrifugation at 9,000 rpm for 20 min followed by 0.22-μm membrane filtration. The CFCS was heated up to 65 °C for 20 min for protease inactivation. The antimicrobial substance production during the *P. alvei* AN5 growth cycle was determined by measuring total activity in arbitrary units (AU), defined as the reciprocal of the highest dilution of CFCS yielding clear inhibition zones against the indicator microorganism [19]. In this study, *Escherichia coli* ATCC 29522 was used as the indicator strain during purification and characterization of the antimicrobial compound.

### Determination of antimicrobial activity

Antimicrobial activity was assessed by agar well diffusion assays as described [6] with some modifications. Briefly, 100 μl of each test strain (OD_600_ = 0.1) was mixed with 20 ml of TSA or NA before being solidified and cast in a Petri dish. A total of three circular wells were cut with diameters of 6 mm and filled with 100 μl of CFCS obtained after 20 h of cultivation. Plates were incubated at 4 °C for 8 h to allow absorption and were further incubated at 37 °C for 24 h. Tests were carried out in triplicate and averages of inhibition zones were reported.

### Purification of the antimicrobial compound

Cell-free culture supernatant was subjected to ultra-filtration using 5, 10 and 30 kDa molecular weight cut-offs (MWCO) (Sigma Aldrich, Germany). Four fractions were obtained and tested against the indicator strain. The CFCS was then subjected to ascending ammonium sulfate saturation at 50, 60, 70 and 80 %. Pellets were obtained by centrifugation at 9,000 rpm and were resuspended in 10 ml of phosphate buffered saline (PBS), pH 7.2. The active fraction with the lowest saturated percentage was subjected to manual column cation exchange chromatography using SP-Sepharose fast flow (Sigma Aldrich, Sweden); equilibration was carried out using 5 mM of ammonium acetate at pH 5. The sample was fractionated using the same buffer with a linear gradient derived from 50 to 1,150 mM NaCl at 100-mM intervals. The active fraction was then further purified using gel filtration chromatography Sephadex G-25 fine (Sigma Aldrich, Germany) and eluted with buffer containing 50 mM sodium phosphate and 150 mM NaCl at pH 7.0. Fractions were collected, concentrated using a freeze dryer and then tested for antimicrobial activity against the indicator strain. The total activity of the purified active fraction was determined by measuring the total activity as described previously [19]. The molecular weight and sample purity were checked using 16.5 % tricine SDS-PAGE as described by Schägger [27, 28]. The gel was cut into vertical parts and one half of the gel was stained with coomassie blue R-250, while the other was overlaid on (0.7 %) soft NA containing fresh overnight culture of the indicator strain, as described [2].

### Infrared spectroscopy (IR) of the antimicrobial peptide

Purified antimicrobial peptide was lyophilized and subjected to Fourier transform infrared (FT-IR) spectroscopy (Thermo scientific NICOLET 6700, Japan) in order to determine the functional groups and chemical bonds.

### Identification of the antimicrobial peptide

The purified sample was further analyzed by integrated nano liquid chromatography electrospray ionization mass spectroscopy (LC/ESI–MS) (Waters, UK). Nano ultra performance liquid chromatography (UPLC) separation was performed with a nano ACQUITY@ UPLC system, equipped with a Symmetry C18 5 μm, 20 mm × 180 μm pre-column and BEH C18 1.7 μm, 20 cm × 75 μm, analytical reversed-phase column. Following the manufacturer’s instructions, 500 ng of sample was initially loaded and transferred with 0.1 % of aqueous formic acid solution to the pre-column at a 15 μl min^−1^ flow rate for 3 min. The mobile phase consisted of 0.1 % formic acid in water (Solvent A) and 0.1 % formic acid in acetonitrile (Solvent B). The sample was separated with a gradient of 1–40 % of solvent B in solvent A for 90 min at a flow rate of 3 μl min^−1^, followed by a 3-min rinse with 85 % of solvent B in solvent A. The column was re-equilibrated to the initial conditions for 20 min. The total cycle time from injection to injection was 120 min. The eluted sample was directly exposed to positive electrospray ionization performed using capillary and cone voltages at 3 kV and 20 V, while the source temperature was maintained at 80 °C. Collision energies were set at 4 V and 15–40 V for both MS and MS/MS. De novo peptide sequencing was performed by data directed analysis (DDA), where the only input required was the intensity threshold to trigger MS/MS acquisition and the MS/MS acquisition time. During the course of MS/MS acquisition, the collision energy was ramped up with a pre-defined collision energy profile, maximizing data quality and information while minimizing user input and the time required. Deisotoping and database searching of the fragmented spectra was automatically performed using the Q-TOF premier system and ProteinLynx Global SERVER v 2.4 software.

### Synthesis of the antimicrobial peptide and minimum inhibitory concentrations (MICs)

The resulting amino acid sequence was sent for peptide synthesis service at Firstbase Laboratories Sdn. Bhd., Malaysia. The obtained peptide was then tested for antimicrobial activity against *E. coli* ATCC 29522. The minimum inhibitory concentrations (MICs) were determined according to the National Committee for Clinical Laboratory Standards (NCCLS). The concentrations of the synthesized antimicrobial peptide AN5-1 ranged from 0.25 to 128 μg ml^−1^. The lowest concentration yielding a clear inhibition zone was reported as the MIC for each test strain.

### Mode of antimicrobial peptide action

Mode of action was screened against four selected test strains: *S. marcescens*, *E. coli* ATCC 29522, *S. aureus* and *B. cereus* ATCC 14579. They were grown in 10 ml of TSB, and the purified antimicrobial peptide at total activity of 80 AU ml^−1^ was added during the early exponential phase. Changes in turbidity at 600 nm were measured at 1-h intervals and compared with control test strains grown in TSB without antimicrobial peptide [15]. The selected test strains were harvested after incubation with peptide AN5-1, re-cultivated on fresh NA and the growth activity was detected. The inhibitory activity (I.A.) of ACE was measured as follows [21]:

$$ {\text{I}}.{\text{A}}. = 100-100 \times \frac{{{\text{OD}}\,600 \left( a \right) }}{{ {\text{OD}}\,600 \left( b \right)}}, $$

where (*a*) is culture containing antimicrobial peptide AN5-1 and (*b*) control without the antimicrobial peptide AN5-1.

### Stability of the antimicrobial peptide

The stability of the purified antimicrobial peptide (80 AU ml^−1^) was determined by exposing it to different ranges of temperature, pH values and chemicals. For temperature stability measurements, the sample was exposed to various temperatures (40–110 °C at intervals of 10 °C) for over 30 min. For pH stability, the samples were incubated at various pH values (2–13 at intervals of 1.0 pH unit) using 0.5N NaOH and 0.5N HCl, followed by 2 h of incubation at 25 °C and then neutralized to their original pH prior to testing antimicrobial activity [5]. Chemical effects were evaluated by incubating the sample with 1 % SDS (w/v), 10 mM EDTA (Vivantis, Malaysia) and 1 % urea (w/v) (Sigma Aldrich, USA) for 1 h at 37 °C, followed by centrifugation at 10,000 rpm for 5 min. The supernatants were tested for antimicrobial activities [15].

## Results

### Antimicrobial compound production during growth cycle

**Fig. 1:**
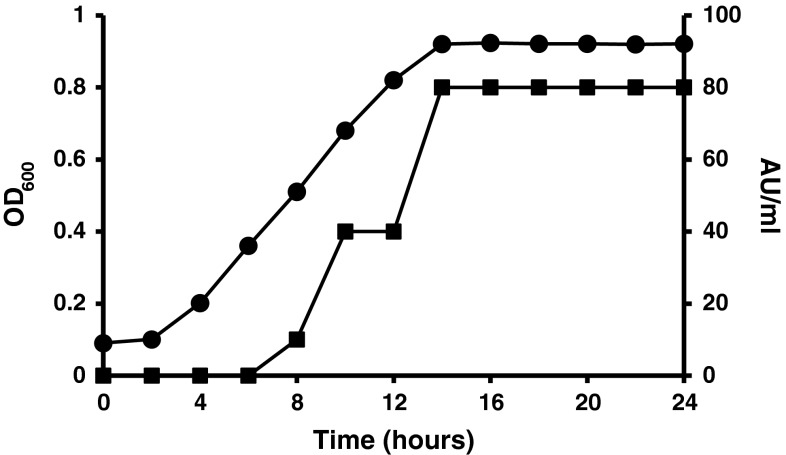
Profile of *Paenibacillus alvei* AN5 growth and antimicrobial peptide production in Modified Landy Medium at 30 °C, 150 rpm in 2 h intervals, absorbance at 600 nm (*filled circle*), and antimicrobial peptide production (AU ml^−1^) determined by micro-dilution assay (*filled square*)

Figure 1 shows the relationship between *P. alvei* AN5 growth and antimicrobial compound production in modified Landy medium. Antimicrobial compound production started in the middle of the exponential phase, where it reached the highest levels of 80 AU ml^−1^ during the early stationary phase. There was no decline in the antimicrobial compound production levels within 24 h of cultivation.

### Screening of the antimicrobial activity

Cell Free Culture Supernatant (80 AU ml^−1^) obtained after 20 h of cultivation inhibited the growth of *Staphylococcus aureus*, *Lactobacillus plantarum* ATCC 8014*, Lactococcus lactis* ATCC 11454, *Bacillus subtilis*, *B. cereus* ATCC 14579, *Bacillus anthracis*, *B. licheniformis*, *Bacillus flexus*, *E. coli* ATCC 25922, *Salmonella enteritidis* ATCC 13076 and *Serratia marcescens.* However, there was no activity observed against *Pseudomonas aeruginosa*, *Streptococcus agalactiae* ATCC 12386*, Staphylococcus epidermidis* and Methicillin Resistance *Staphylococcus aureus,* MRSA (Table 1).

### Identification of the antimicrobial peptide

**Fig. 2:**
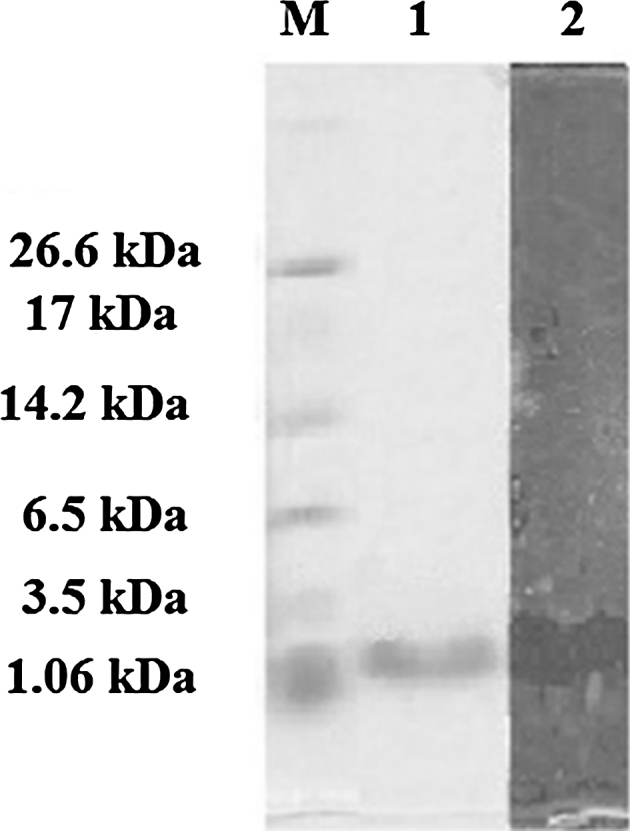
Tricine SDS-PAGE supplemented with glycerol for purified antimicrobial peptide AN5-1 produced by *Paenibacillus alvei* AN5. *Lane 1* color marker ultra-low range (M.W. 1.06–26.6) KDa (Sigma, USA), *lane 2* purified AN5-1 stained with Coomassie blue, *lane 3* bacteriocin assay overlying on soft agar shows inhibitory activity of the active peptide against *E. coli* ATCC 29522

**Fig. 3:**
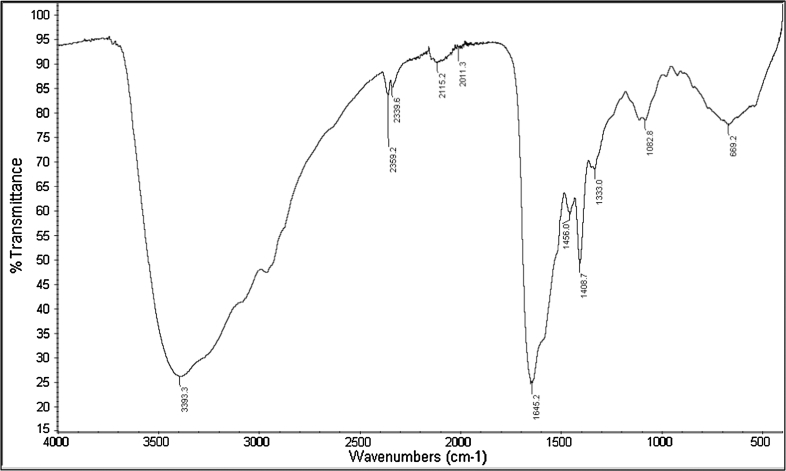
Fourier transform infrared (FT-IR) of purified antimicrobial peptide AN5-1

**Fig. 4:**
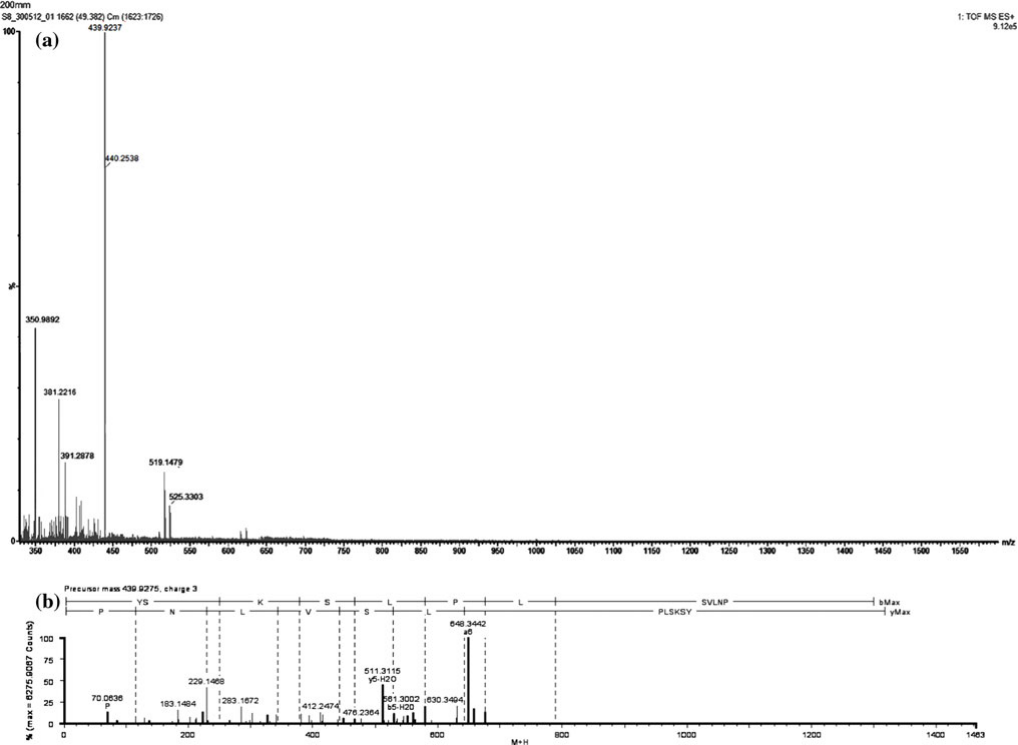
Mass spectroscopy of purified antimicrobial peptide AN5-1, **a** ESI spectra of purified antimicrobial peptide AN5-1 obtained from UPLC shows the single band has three charges of molecular mass 439.9275*m/z* ionized by ESI with overall molecular mass 1,316.7340 Da. **b** MS/MS spectra of purified antimicrobial peptide AN5-1 performed automatically using ProteinLynx Global SERVER v 2.4 software based on Q-TOF premier system

Cell Free Culture Supernatant was fractionated using 30-, 10- and 5-kDa (MWCO) filters. Supernatant obtained below 5 kDa was the only active fraction against the indicator strain. The CFCS was subjected to ascending ammonium sulfate precipitation from 50 to 80 % in 10-unit intervals. The lowest active saturated percentage was 70 %, which was further purified using SP-Sepharose fast flow. The active fraction was eluted with 750 mM of NaCl in 5 mM ammonium acetate and was further purified using gel filtration chromatography, resulting in six fractions where only the first elution was active against the indicator strain. The purity of the sample was checked using Tricine SDS-PAGE, where a single band with a molecular mass estimated to be ~1.5 kDa was observed. This band showed antimicrobial activity on soft agar against *E. coli* ATCC 29522 (Fig. 2). The purified antimicrobial compound was lyophilized and subjected to FT-IR, which showed major peaks at 3,393.3, 1,645.2 and 1,408.7 cm^−1^ (Fig. 3). Both the FT-IR and SDS-PAGE findings demonstrated the protein nature of the active antimicrobial compound found in CFCS. The purified antimicrobial peptide was further analyzed using LC–ESI/TOF–MS. The MS analysis indicated the presence of a peptide 1,316.7340 Da in size (Fig. 4a). The TOF–MS/MS spectrum consisted of a series of *y* and *b* ions with several ion fragments. Molecular weight subtractions for the resulting *m/z* values were automatically performed. The amino acid sequence was deducted by interpreting the ESI–MS/MS spectra displaying the fragments of *m/z* 116.1 (*y*1), *m/z* 136.1 (*a*1), *m/z* 230.1 (*y*2), *m/z* 251.1 (*b*2), *m/z* 343.2 (*y*3), *m/z* 379.2 (*b*3), *m/z* 442.3 (*y*4), *m/z* 466.2 (*b*4), *m/z* 529.3 (*y*5), *m/z* 579.3 (*b*5), *m/z* 676.3 (*b*6) and *m/z* 789.4 (*b*7) (Fig. 4b), which suggested the amino acid sequence of AN5-1 to be Y-S-K-S-L-P-L-S-V-L-N-P. The peptide sequence was then compared with the NCBI and APD databases, with the results suggesting a novel antimicrobial peptide that displayed low similarity to existing antimicrobial peptides (Table 2). Antimicrobial peptide AN5-1 was registered in UniProt under the accession number B3EWQ6. In order to verify the antimicrobial activity was due to the antimicrobial peptide AN5-1, the determined amino acid sequence was synthesized and further tested for antimicrobial activity. The purity was checked by the manufacturer using HPLC, and resulted in >90 % which is acceptable for biological activity tests according to the manufacturer's instructions. The synthesized peptide AN5-1 was tested against *E. coli* ATCC 29522, and the resulting inhibition zones were 17 mm similar to the one obtained from CFCS, which confirms the active structure of the small antimicrobial peptide AN5-1.

**Table 2:** Comparison of antimicrobial peptide AN5-1 with other antimicrobial peptides existing in APD

Peptide or protein	Sequence	Source	Similarity (%)	References
AN5-1	YSKSLPLSVLNP	*P. alvei AN5*	–	This study
Temporin H	LSPNLLKSLL	*Rana temporaria*	38.46	[29]
Temporin-1Cd	FLPFLASLLSKVL	*Rana clamitan*	37.5	[11]
Odorranain-A-OA1	VVKCSYRLGSPDSQCN	*Odorrana andersonii*	35.29	[32]
Alyteserin-2b	ILGAILPLVSGLLSNKL	*Odorrana andersonii*	35.29	[8]
Temporin 1Ja	ILPLVGNLLNDLL	*Odorrana andersonii*	35.29	[13]

### Characterization and MICs of the antimicrobial peptide

**Fig. 5:**
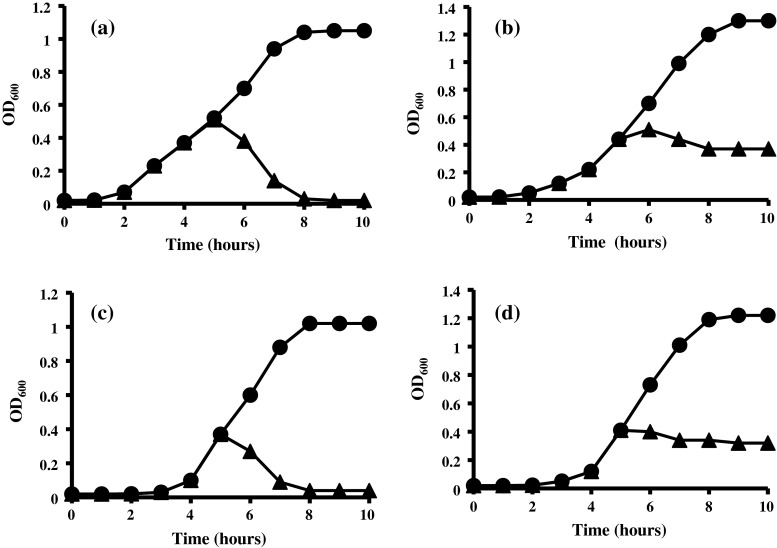
Effects of 80 AU ml^−1^ of purified antimicrobial peptide AN5-1 produced by *P. alvei* AN5 on early exponential growth phase against target strains: **a** *E. coli* ATCC 29522, **b** *S. marcescens*, **c** *S. aureus* and **d** *B. cereus* ATCC 14579, in the absence (*filled circle*) and presence (*filled triangle*) of antimicrobial peptide AN5-1. The bacterial growth was measured by means of optical density at 600 nm (OD_600_)

The MICs of the synthesized antimicrobial peptide AN5-1 were assessed against test strains and the resulting MICs are reported in Table 1. Antimicrobial peptide AN5-1 has exhibited a strong antimicrobial activity against Gram-negative bacterial strains of *S. enteritidis* ATCC 13076 and *E. coli* ATCC 29522 with MICs values of 4 and 8 μg ml^−1^, where *B. anthracis*, *B. licheniformis* and *L. lactis* ATCC 11454 showed less sensitivity toward AN5-1 with MICs values of 128 and >128 μg ml^−1^. To determine the mode of AN5-1 action, four selected strains of *S. marcescens*, *E. coli* ATCC 29522, *S. aureus* and *B. cereus* ATCC 14579 were grown separately in NB and 80 AU ml^−1^ of AN5-1 were added after 5 h of cultivation. The addition of AN5-1 to *E. coli* ATCC 29522 and *S. aureus* led to a rapid decrease in cell density (Fig. 5a, c), ending with complete growth inhibition. However, both *S. marcescens* and *B. cereus* ATCC 14579 showed less sensitivity towards AN5-1, showing only a slight decrease in cell density or remaining static (Fig. 5b, d). The selected test strains were collected after incubation with peptide AN5-1 and re-cultivated on fresh NA, there was no growth observed confirming the bactericide action rather than bacteriostatic. The inhibitory activities were measured at 1-h intervals after AN5-1 addition, where *S. aureus* and *E. coli* ATCC 29522 showed a relatively higher inhibitory effect (96.07 and 98.05 %) compared to *S. marcescens* (71.52 %) and *B. cereus* ATCC 14579 (72.77 %) (Table 3). The AN5-1 showed stability at different temperatures within the range of 40–90 °C using *E. coli* ATCC 29522 as the indicator strain. The activity of AN5-1 declined at 100 °C and was completely absent at 110 °C, indicating that the AN5-1 peptide was thermo-labile. In addition, AN5-1 retained its activity within the range of pH 2 to 12 and was completely inactivated at pH 1 and 13. The AN5-1 was also not affected by chemicals such as SDS, urea and EDTA (Table 4). Storage of AN5-1 at −20 °C did not affect the antimicrobial property for 120 days. Storage at 4 and 37 °C showed a declining pattern of antimicrobial property after 85 and 20 days, respectively.

**Table 3:** Inhibitory activities, percentage of antimicrobial peptide AN5-1 at 80 AU ml^−1^ against selective target cells

Target cells	Inhibitory activities (%)				
	1 h	2 h	3 h	4 h	5 h
*E. coli* ATCC 29522	45.71	85.1	97.1	98.05	98.05
*S. marcescens*	27.1	55.55	69.1	71.5	71.5
*S. aureus*	55	89.7	96.07	96.07	96.07
*B. cereus* ATCC 14578	45.02	66.33	71.42	72.77	72.77

**Table 4:** Stability of purified antimicrobial peptide AN5-1 (80 AU ml^−1^) against temperature, pH and chemicals using *E. coli* ATCC 13076 as indicator strain

Stability	Inhibition zones diameter
Temperature (°C)	
40	+++
50	+++
60	+++
70	+++
80	+++
90	+++
100	++
110	–
pH values	
1	–
2	+
3	+++
4	+++
5	+++
6	+++
7	+++
8	+++
9	+++
10	+++
11	+++
12	++
13	–
Chemicals	
SDS	+++
EDTA	+++
Urea	+++

–, no inhibition zones; +, zone diameter 1–11 mm; ++, zone diameter 12–16 mm; +++, zone diameter 16–21 mm; ++++, zone diameter ≥22 mm

## Discussion

In this study, a novel antimicrobial compound was isolated from the CFCS of *P. alvei* AN5 during the early exponential growth phase. It has been reported that bacterial strains freshly isolated from their natural habitats contain the gene needed for antimicrobial peptide production [26]. Under laboratory conditions, bacterial strains grow as a monoculture with no microbial competitors and less stress, which make antimicrobial peptide production unnecessary in the culture [26]. The antimicrobial compound purified in this study showed a wide bactericidal inhibitory spectrum against both Gram-positive and negative strains (Table 1). Purification of CFCS was carried out in three steps, salting out, cation exchange chromatography and gel filtration chromatography. The proteinaceous nature of the purified antimicrobial compound was confirmed by the existence of N–H stretching (amide A) and C=O stretching (amide I) at 3,393.3 and 1,645.2 cm^−1^ ,respectively [16]. Meanwhile, absorbance at 1,408.7 cm^−1^ corresponded to the C=O stretching (symmetric) of COO^−^ [18]. Further analysis using LC–ESI/TOF–MS demonstrated a molecular weight of 1,316.7340 Da, which was derived from a triple-charged single ion peak ionized by ESI as the ionization source (Fig. 4a). Based on the automatic calculation using data analysis software (PLGS), DDA analysis suggested that the primary structure of the antimicrobial peptide AN5-1 was Y-S-K-S-L-P-L-S-V-L-N-P (Fig. 4b).

The sequence obtained was matched against the NCBI swissprot database, resulting in 83 % partial identity with a putative uncharacterized protein (accession number: B3EFZ9) isolated from *Chlorobium limicola* (strain DSM 245/NBRC 103803). To date, protein B3EFZ9 has not been reported to possess antimicrobial activity. Meanwhile, comparison with the APD database resulted in 38.46 % similarity with Temporin H isolated from *Rana temporaria* [29], 37.5 % similarity with Temporin-1Cd isolated from *Rana clamitan* [11], and only 35.29 % similarity with Odorranain-A-OA1, Alyteserin-2b and Temporin 1Ja isolated from *Odorrana andersonii*, the European midwife toad and *Rana japonica*, respectively [8, 13, 32].

It has been reported that low molecular mass antimicrobial peptides produced by Gram-positive bacteria have bactericidal action [14]. As previously reported in 2005, antimicrobial peptides interact with the bacterial outer membrane and pass through, causing pore formations that may lead to cell lysis. However, the mechanism of both pore formation and cell lysis is not well understood and often based on the metabolic activity of the target cell [3, 17]. Both positive charge and hydrophobic amino acids content of the antimicrobial peptides play a major role in the interactions with the negatively charged membrane of the target cell [30, 31]. In this study, we speculated that the antimicrobial peptide AN5-1 with a positive charge and four hydrophobic amino acids residues of one valine (V) and three leucine (L), was likely to interact with the target cell membrane, causing pore formations that led to cell lysis such as *E. coli* ATCC 29522 and *S. aureus* (Fig. 5a, c). On the other hand, the steady OD_600_ measurements after addition of AN5-1 to both *S. marcescens* and *B. cereus* ATCC 14579 (Fig. 5b, d), were an evidence of a bactericidal with no cell lysis action, suggesting that the formed pores led to either damage in the macronutrient synthesis such as nucleic acids and proteins, or lipid modifications in the membrane bilayer rather [30]. The high stability against heat and pH ranges are two major industrial characteristics of AN5-1 that make it a promising candidate as a food preservative, compared with nisin, whose maximum acidic stability reaches a pH value of 2 [4].

## Conclusion

In the present study, we purified and characterized a new antimicrobial peptide (AN5-1) secreted into the cultivation medium of *P. alvei* AN5. Peptide AN5-1 showed remarkable stability over a wide temperature and pH range. The AN5-1 also displayed a bactericidal broad spectrum of targets against both Gram-positive and -negative bacteria. However, further studies are in process in our laboratory investigating the mechanism of AN5-1 action and target cell morphological changes.

## Abbreviations

NB: Nutrient broth
NA: Nutrient agar
TSB: Tryptic soy broth
TSA: Tryptic soy agar
CFCS: Cell free culture supernatant
AU: Arbitrary unit
